# Supplementary material for: TRAF3IP3 negatively regulates cytosolic RNA induced anti-viral signaling by promoting TBK1 K48 ubiquitination
Source: Nat Commun. 2020 May 4;11:2193. doi: 10.1038/s41467-020-16014-0 (PMC7198545; doi:10.1038/s41467-020-16014-0)
Supplement: Supplementary file 2 — Supplementary Information [file 41467_2020_16014_MOESM2_ESM.pdf]

Supplementary information for

TRAF3IP3 negatively regulates cytosolic RNA induced anti-viral signaling by promoting TBK1 K48 ubiquitination.

Meng Deng, Jason W. Tam, Lufei Wang et al.

This file contains Supplementary Figure 1-7 and Supplementary Table 1.

a

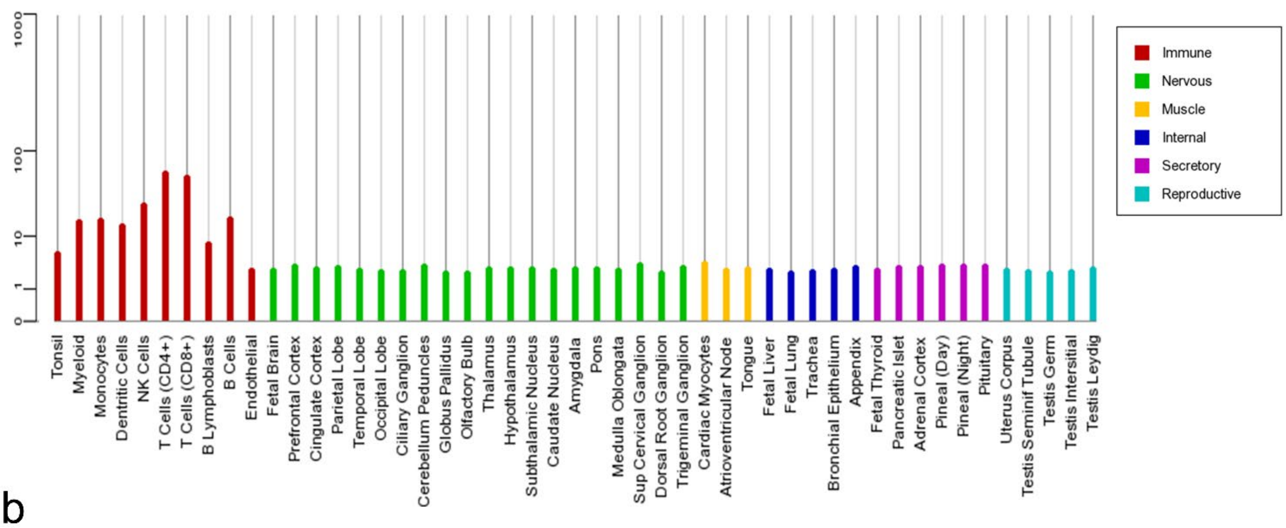
1000
100
10
1
0
Immune
Nervous
Muscle
Internal
Secretory
Reproductive
Tonsil
Myeloid
Monocytes
Dentritic Cells
NK Cells
T Cells (CD4+)
T Cells (CD8+)
B Lymphoblasts
B Cells
Endothelial
Fetal Brain
Prefrontal Cortex
Cingulate Cortex
Parietal Lobe
Temporal Lobe
Occipital Lobe
Ciliary Ganglion
Cerebellum Peduncles
Globus Pallidus
Olfactory Bulb
Thalamus
Hypothalamus
Subthalamic Nucleus
Caudate Nucleus
Amygdala
Pons
Medulla Oblongata
Sup Cervical Ganglion
Dorsal Root Ganglion
Trigeminal Ganglion
Cardiac Myocytes
Atrioventricular Node
Tongue
Fetal Liver
Fetal Lung
Trachea
Bronchial Epithelium
Appendix
Fetal Thyroid
Pancreatic Islet
Adrenal Cortex
Pineal (Day)
Pineal (Night)
Pituitary
Uterus Corpus
Testis Seminif Tubule
Testis Germ
Testis Intersitial
Testis Leydig

b

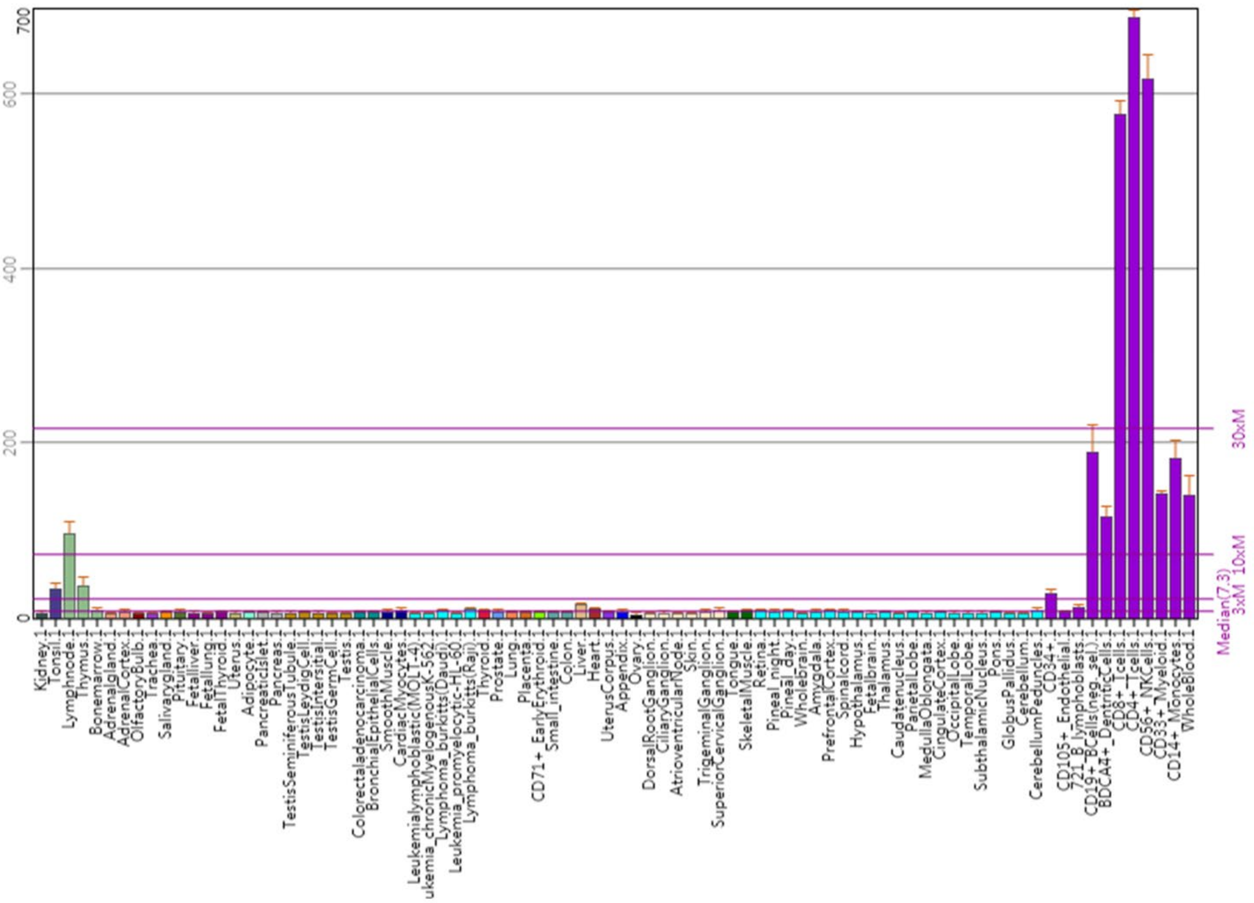
700
600
400
200
0
30xM
10xM
3xM
Median(7.3)

c

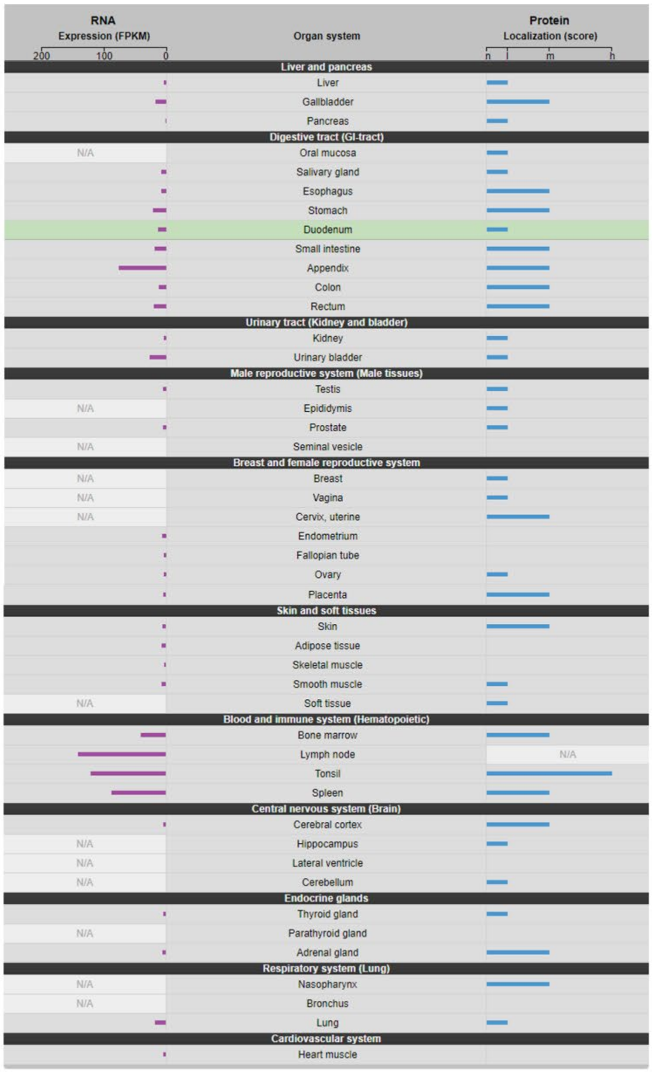
RNA
Expression (FPKM)
200
100
0
Organ system
Protein
Localization (score)
n
l
m
h
Liver and pancreas
Liver
Gallbladder
Pancreas
Digestive tract (GI-tract)
N/A
Oral mucosa
Salivary gland
Esophagus
Stomach
Duodenum
Small intestine
Appendix
Colon
Rectum
Urinary tract (Kidney and bladder)
Kidney
Urinary bladder
Male reproductive system (Male tissues)
Testis
N/A
Epididymis
Prostate
N/A
Seminal vesicle
Breast and female reproductive system
N/A
Breast
N/A
Vagina
N/A
Cervix, uterine
Endometrium
Fallopian tube
Ovary
Placenta
Skin and soft tissues
Skin
Adipose tissue
Skeletal muscle
Smooth muscle
N/A
Soft tissue
Blood and immune system (Hematopoietic)
Bone marrow
Lymph node
N/A
Tonsil
Spleen
Central nervous system (Brain)
Cerebral cortex
N/A
Hippocampus
N/A
Lateral ventricle
N/A
Cerebellum
Endocrine glands
Thyroid gland
N/A
Parathyroid gland
Adrenal gland
Respiratory system (Lung)
N/A
Nasopharynx
N/A
Bronchus
Lung
Cardiovascular system
Heart muscle

d

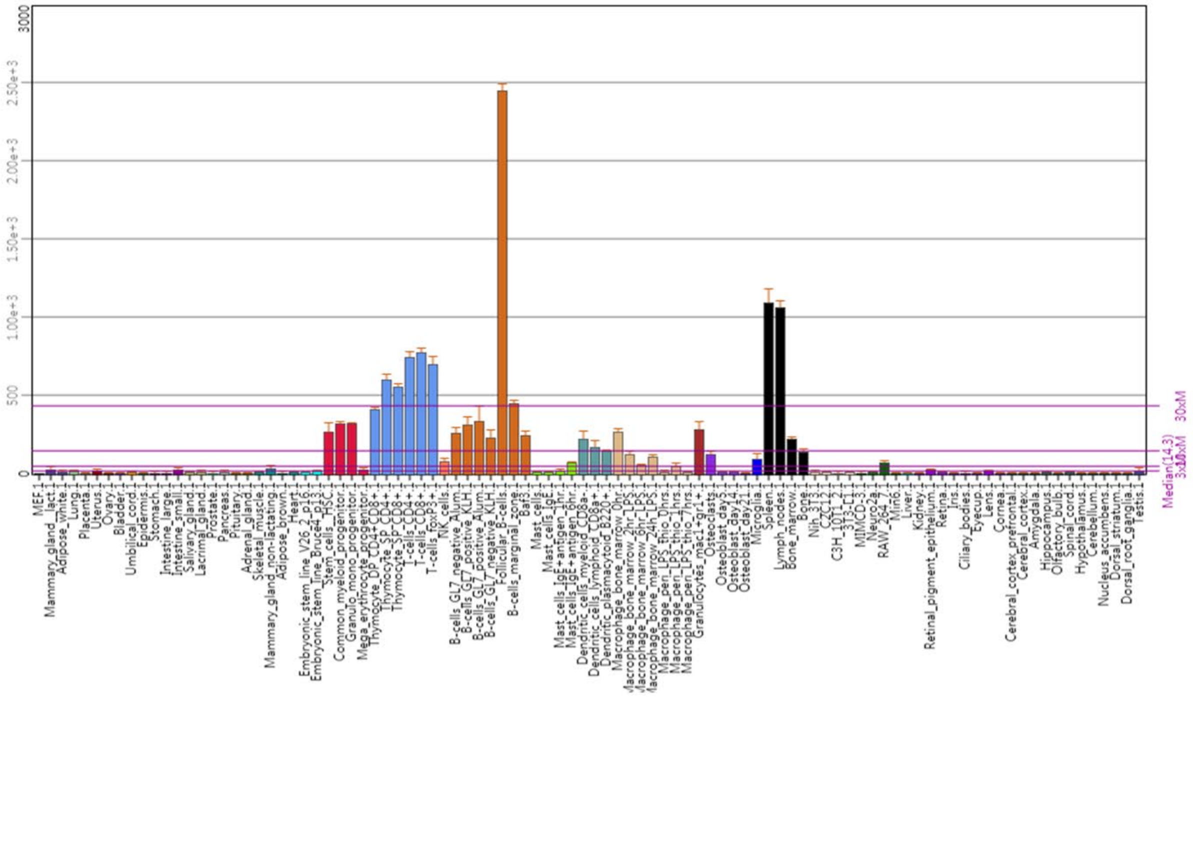
3000
2.50e+3
2.00e+3
1.50e+3
1.00e+3
500
0
30xM
10xM
3xM
Median(14.3)

Supplementary Fig. 1. TRAF3IP3 expression profiling in human and mice tissues.

a *TRAF3IP3* mRNA expression in normal human tissues (normalized intensities) from GeneCards.

b *TRAF3IP3* mRNA expression in normal human tissues (normalized intensities) from BioGPS.

c *TRAF3IP3* mRNA and protein expression in normal human tissues (normalized intensities) from Human Protein Atlas.

d *Traf3ip3* mRNA expression in normal mouse tissues (normalized intensities) from BioGPS.

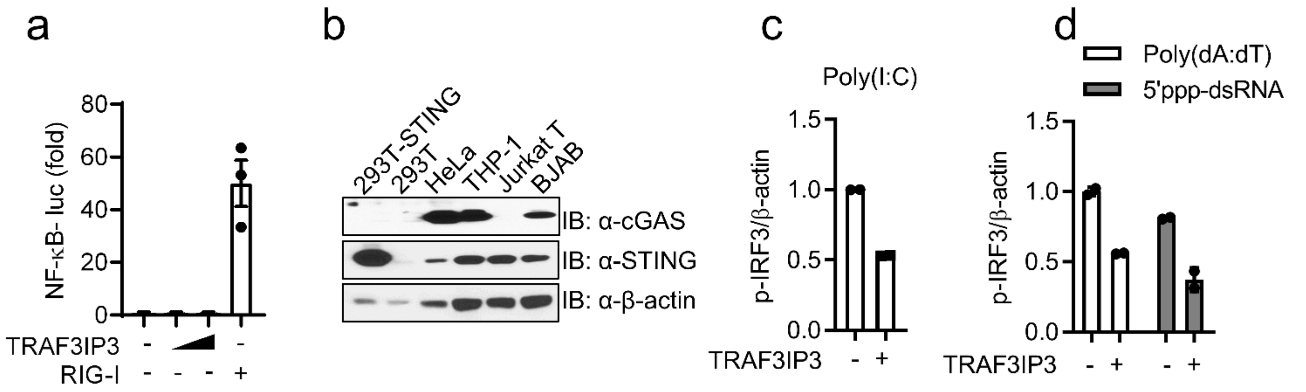

Supplementary Fig. 2. The role of TRAF3IP3 in IFN-I signaling.

a Luciferase assay in HEK293T cells transfected with NF-κB reporter, along with empty vector, increasing doses of the Myc-TRAF3IP3 vector (wedge), or Flag-RIG-I vector. *Renilla* luciferase was used as internal control.

b Immunoblotting using indicated cell lines. HEK293T cells transfected with Flag-STING serves as the positive control.

c Densitometry of Fig. 1i using the sum of p-IRF3 intensity against the sum of β-actin intensity

d Densitometry of Fig. 1j.

Data in a and b are one representative of three independent experiments. Data in a are presented as mean ± SEM and data in c and d are presented as mean ± SD. $^{*}p < 0.05$, $^{**}p < 0.01$, $^{***}p < 0.001$.

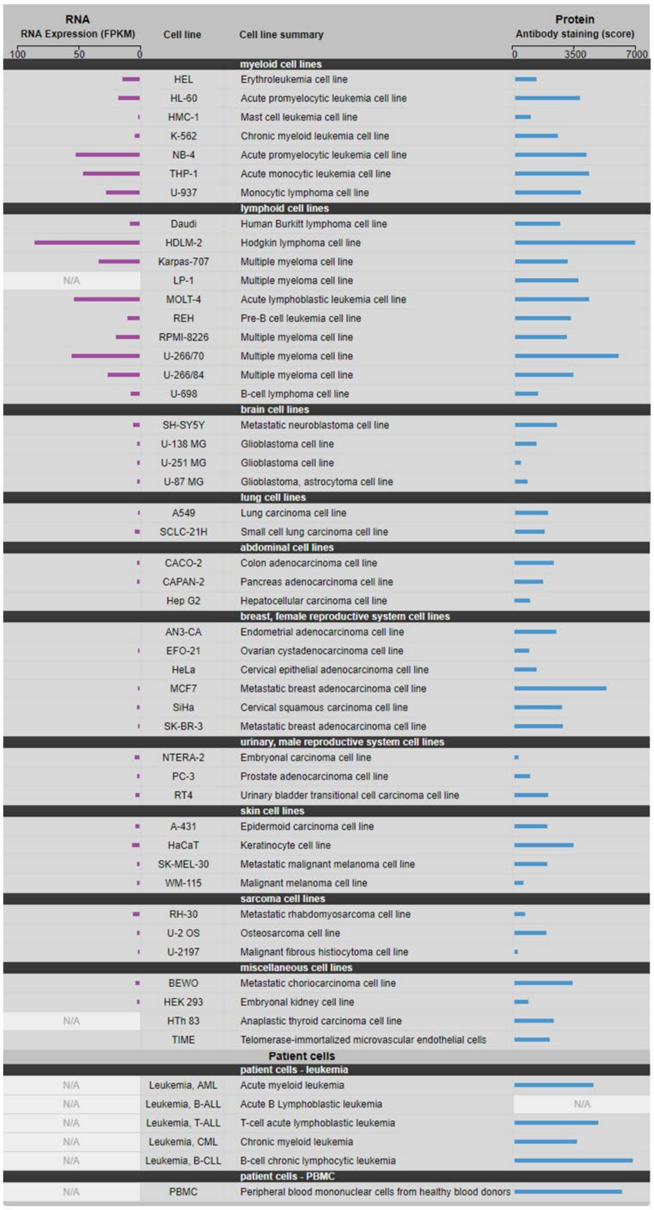

Supplementary Fig. 3. TRAF3IP3 expression profiling in human cell lines.

TRAF3IP3 mRNA and protein expression in human cell lines (normalized intensities) from the Human Protein Altas.

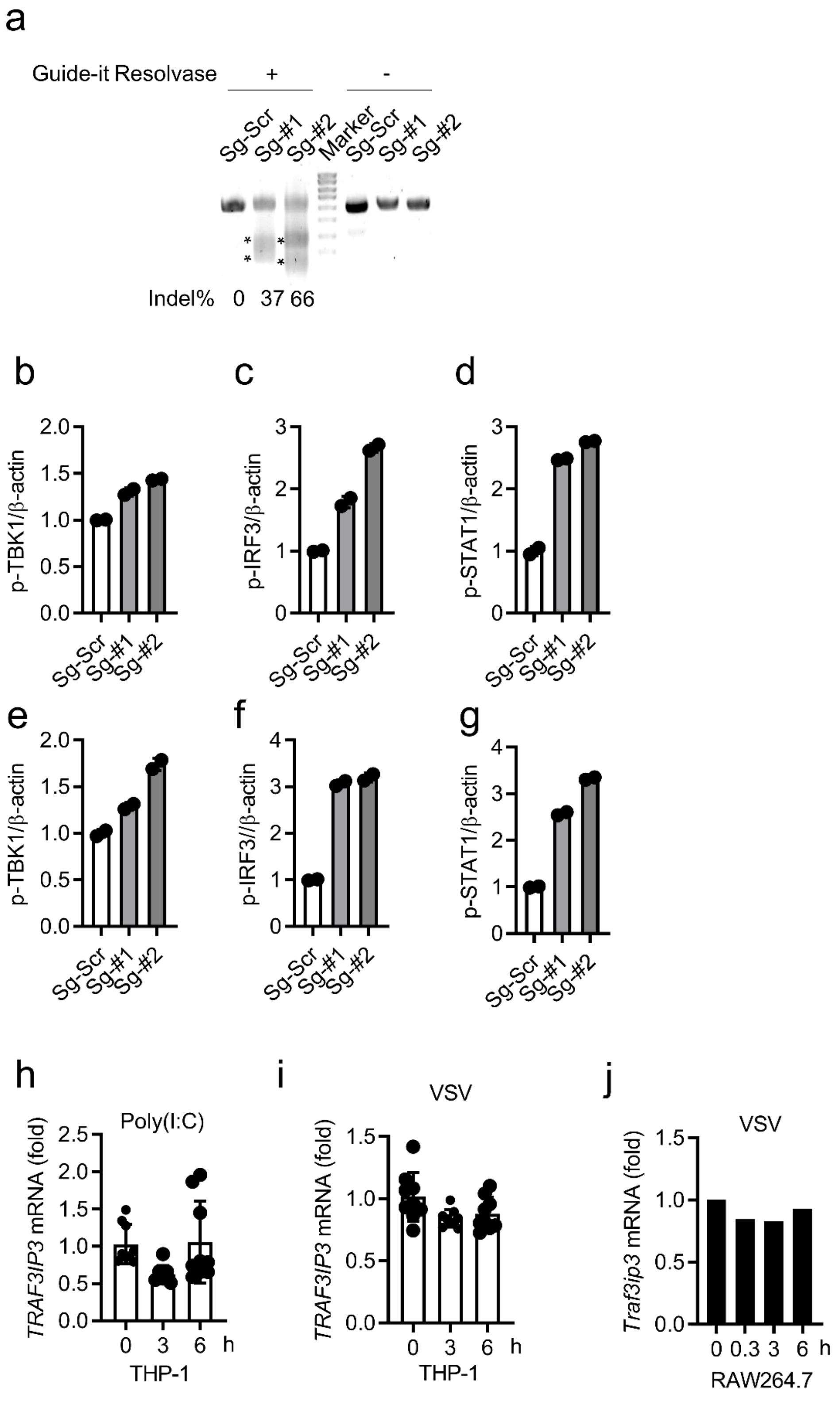

Supplementary Fig. 4. Reduction of TRAF3IP3 potentiates IFN-I signaling induced by cytosolic RNA.

a Two sgRNAs are designed to target the human *TRAF3IP3* loci. Indel frequency was calculated.

Asterisks indicate expected fragment sizes for each locus.

b-d Densitometry of Fig. 2c using the sum of intensity at 0, 3, 6, 9h.

e-g Densitometry of Fig. 2d using the sum of intensity at 0, 3, 6, 9h.

h, i RT-PCR analysis of *TRAF3IP3* mRNA in THP1 cell after transfection of Poly(I:C) (**h**) or infected with VSV (MOI = 1) (**i**). RT-PCR data were normalized to *Actb* mRNA.

j *Traf3ip3* mRNA in RAW264.7 cells after VSV challenge from Infectome Map.

Data are presented as mean ± SD. Data in a, h and i are one representative of three independent experiments.

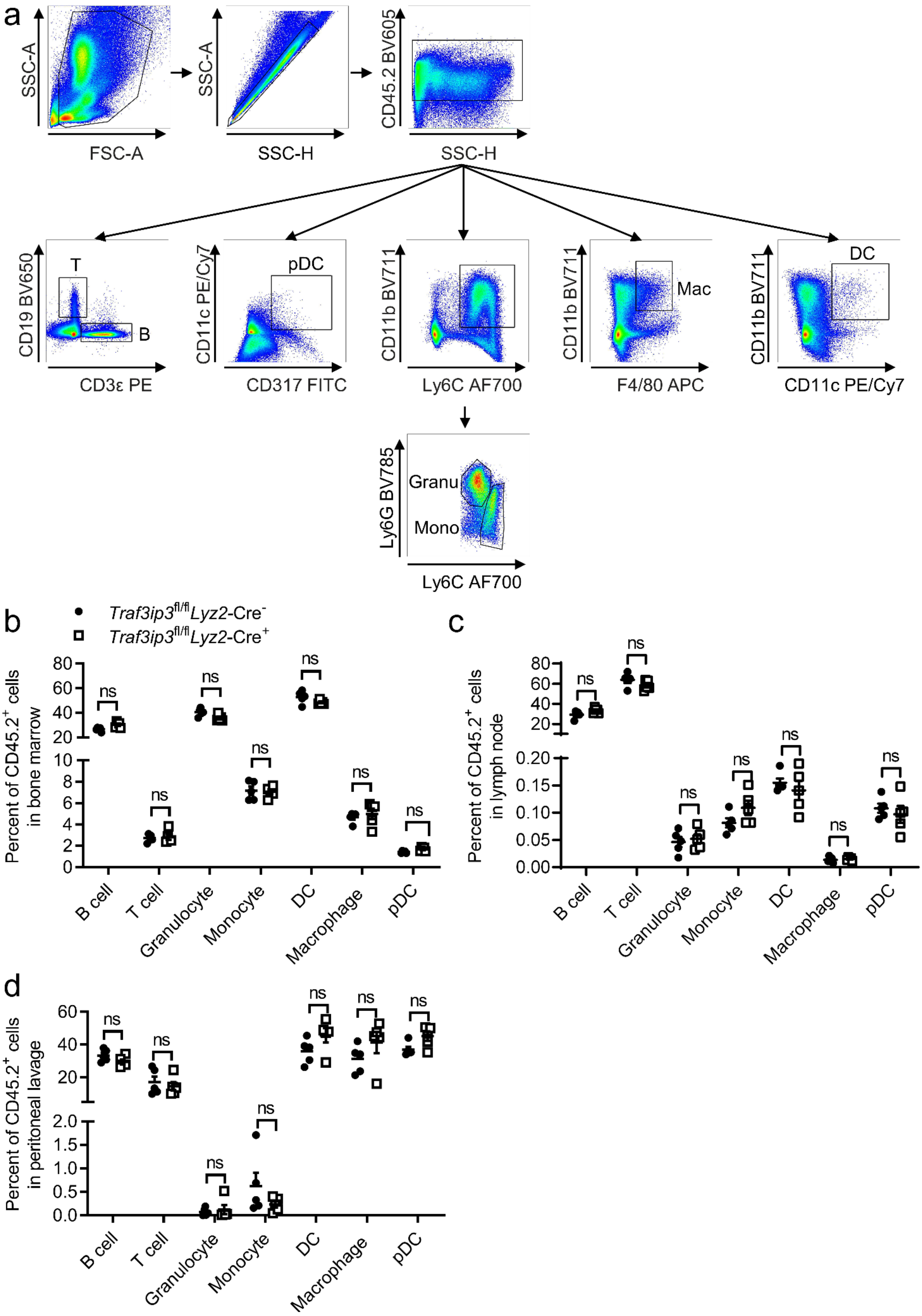
a
SSC-A
FSC-A
SSC-A
SSC-H
CD45.2 BV605
SSC-H
CD19 BV650
T
B
CD3ε PE
CD11c PE/Cy7
pDC
CD317 FITC
CD11b BV711
Ly6C AF700
CD11b BV711
Mac
F4/80 APC
CD11b BV711
DC
CD11c PE/Cy7
Ly6G BV785
Granu
Mono
Ly6C AF700
b
Traf3ip3fl/fl Lyz2-Cre-
Traf3ip3fl/fl Lyz2-Cre+
Percent of CD45.2+ cells in bone marrow
ns
B cell
T cell
Granulocyte
Monocyte
DC
Macrophage
pDC
c
Percent of CD45.2+ cells in lymph node
d
Percent of CD45.2+ cells in peritoneal lavage

Supplementary Fig. 5. Immune development in conditional *Traf3ip3* knockout mice is normal.

a Representative flow-cytometry gating strategy using sample from b.

b-d FACS analysis of immune cells in different tissues from 8-week-old mice of indicated genotype using markers shown for the different cell populations. n=5 biologically independent animals. Data are presented as mean ± SEM and are one representative from two independent experiments. b-d, t-test. ns, not significant.

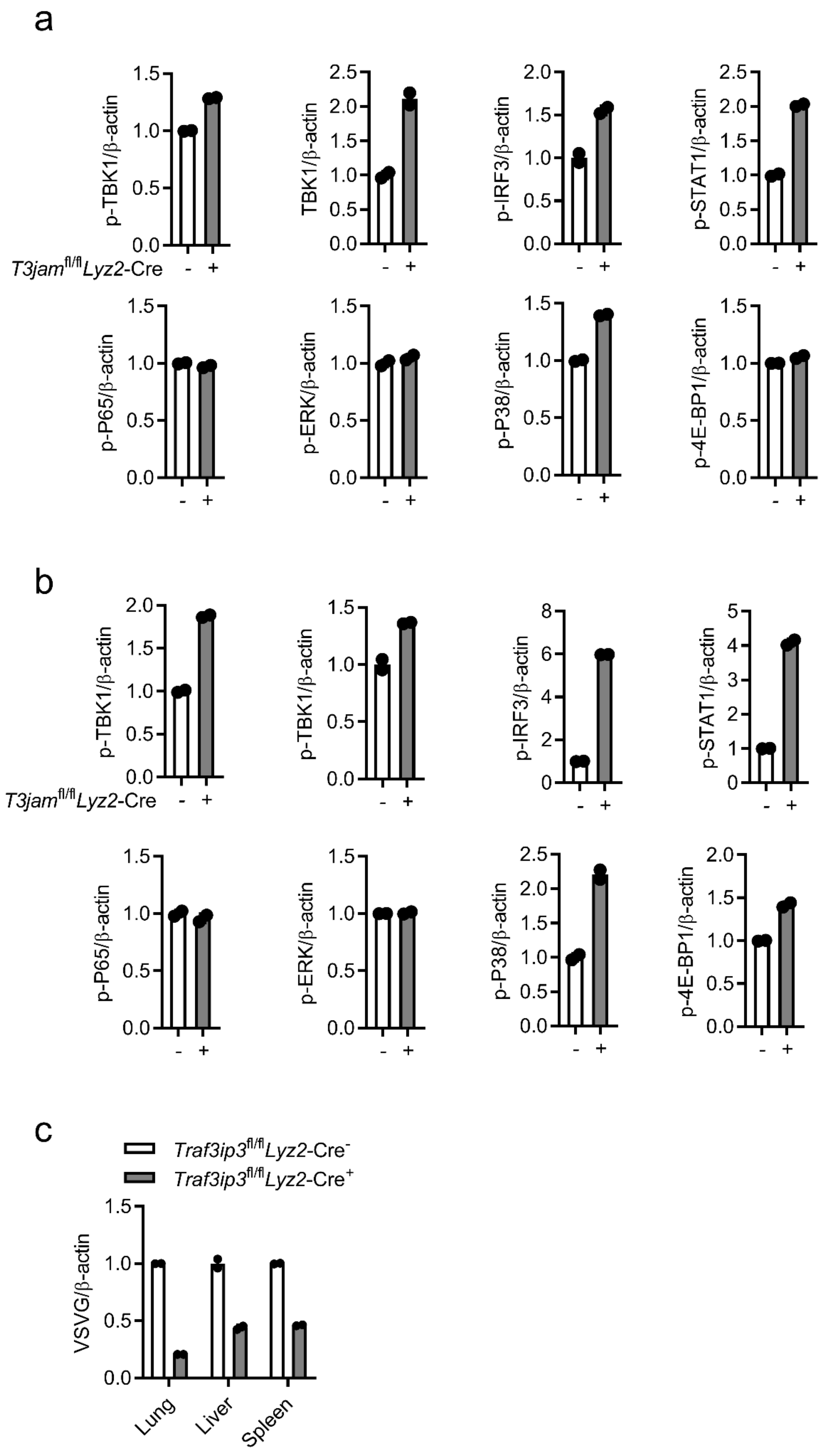
a
p-TBK1/β-actin
TBK1/β-actin
p-IRF3/β-actin
p-STAT1/β-actin
T3jamfl/flLyz2-Cre
- +
p-P65/β-actin
p-ERK/β-actin
p-P38/β-actin
p-4E-BP1/β-actin
b
p-TBK1/β-actin
p-TBK1/β-actin
p-IRF3/β-actin
p-STAT1/β-actin
T3jamfl/flLyz2-Cre
- +
p-P65/β-actin
p-ERK/β-actin
p-P38/β-actin
p-4E-BP1/β-actin
c
Traf3ip3fl/flLyz2-Cre-
Traf3ip3fl/flLyz2-Cre+
VSVG/β-actin
Lung
Liver
Spleen

Supplementary Fig. 6. *Traf3ip3* deficiency potentiates IFN-I signaling induced by cytosolic RNA.

a Densitometry of Fig. 3f.

b Densitometry of Fig. 3g.

c Densitometry of Fig. 7e.

Data are presented as mean ± SD.

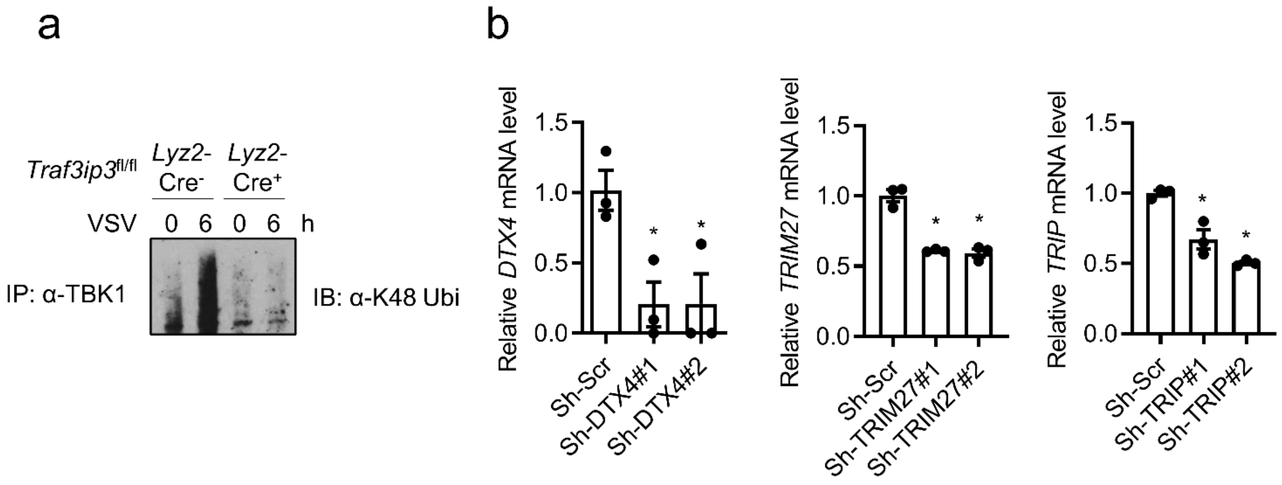

Supplementary Fig. 7. TRAF3IP3 targets TBK1 and TRAF3

a Immunoprecipitation and immunoblotting using *Traf3ip3*$^{fl/fl}$ *Lyz2*-Cre^{-} and *Traf3ip3*$^{fl/fl}$ *Lyz2*-Cre^{+} BMDMs infected with VSV (MOI = 1) for the indicated time. Data are one representative of at least two independent experiments.

b Knockdown efficiency using indicated shRNA in 293T cells. Data are pooled from three independent experiments and presented as mean ± SEM. One-way ANOVA followed by Dunnet post hoc correction. $^{*}p < 0.05$.

Oligonucleotides (5’ - >3’)	
IFNB1-F	CATTACCTGAAGGCCAAGGA
IFNB1-R	CAATTGTCCAGTCCCAGAGG
ACTB-F	AGAGCTACGAGCTGCCTGAC
ACTB-R	AGCACTGTGTTGGCGTACAG
TBK1-F	AGCGGCAGAGTTAGGTGAAA
TBK1-R	CCAGTGATCCACCTGGAGAT
TRAF3IP3-F	TTCTCCCAGAGAGCAGGTGA
*TRAF3IP*3-R	TGGTGTTTGGGTGGCTTCTT
ISG15-F	TCCTGGTGAGGAATAACAAGGG
ISG15-R	GTCAGCCAGAACAGGTCGTC
DTX4-F	GCCACCTTGAATCGTACCAACC
DTX4-R	GGTTGACAGGACTGGACCCATT
TRIM27-F	AGCCTGATCGCTCAGCTAGAAG
TRIM27-R	GGAGGTGTGATCCAAGGTTCAG
TRAIP-F	CGCTGGAAGAACGCAATGCTAC
TRAIP-R	GTGCTTGTTTGGTCTCATCCTGC
Ifnb-F	ATGAGTGGTGGTTGCAGGC
Ifnb-R	TGACCTTTCAAATGCAGTAGATTCA
Actb-F	AGGGCTATGCTCTCCCTCAC
Actb-R	CTCTCAGCTGTGGTGGTGAA
Isg15-F	CACAGTGATGCTAGTGGTAC
Isg15-R	CTTAAGCGTGTCTACAGTCTG
Sg-TRAF3IP3-1-F	CACCGAGCCCACCGGGCCAAGCCAG
Sg-TRAF3IP3-1-R	AAACCTGGCTTGGCCCGGTGGGCTC
Sg-TRAF3IP3-2-F	CACCGCAATGTGACCACTTGCCGCC
Sg-TRAF3IP3-2-R	AAACGGCGGCAAGTGGTCACATTGC
Sg-Scramble-F	CACCGTGCGAATACGCCCACGCGAT
Sg-Scramble-R	AAACATCGCGTGGGCGTATTCGCAC
F (amplicon for Indel)	CCAAGAGTCCTGACTCCCACT
R (amplicon for Indel)	CTCCTTGTCCAGAGAGGGT
siRNA-Scramble	rCrGrUrUrArArUrCrGrCrGrUrArUrArArUrArCrGrCrGrUAT
siRNA-TRAF3IP3 #1	rArGrCrArGrArUrArGrCrArGrGrGrArCrUrUrArCrArGrATG
siRNA-TRAF3IP3 #2	rGrCrArCrUrGrUrCrGrArGrArArGrArGrCrUrGrArArCrCAG
Sh-TRIM27 (TRCN0000006437)	CCGGCAGGGCTGAAAGAATCAGGATCTCGAGATCCTGATTCT TTCAGCCCTGTTTTT
Sh-TRIM27 (TRCN0000006438)	CCGGGAATTAAGAGAGGCTCAGTTACTCGAGTAACTGAGCC TCTCTTAATTCTTTTT
Sh-TRIP (TRCN0000033740)	CCGGCCCAGCATGGTTACTACGAAACTCGAGTTTCGTAGTAA CCATGCTGGGTTTTTG
Sh-TRIP (TRCN0000033741)	CCGGGCAGTGCCTAATTCAGTGGTTCTCGAGAACCACTGAAT TAGGCACTGCTTTTTG
Sh-DTX4 (TRCN0000236607)	CCGGTTAAGGCAGCCGTGGTCAATGCTCGAGCATTGACCAC GGCTGCCTTAATTTTTG
Sh-DTX4 (TRCN0000236608)	CCGGCATTGGCTTTAGCTACGTAATCTCGAGATTACGTAGCTA AAGCCAATGTTTTTG

Supplementary Table 1. Oligonucleotides used in this study.
